# Awareness and Knowledge of the Physical Activity Guidelines and Their Association with Physical Activity Levels

**DOI:** 10.3390/sports12070174

**Published:** 2024-06-25

**Authors:** Ahmed M. Wafi, Saud N. Wadani, Yazan Y. Daghriri, Ali I. Alamri, Abdulrahim M. Zangoti, Ayman A. Khiswi, Elyas Y. Al-Ebrahim, Hemachandran J. Jesudoss, Abdullah A. Alharbi

**Affiliations:** 1 Basic Medical Science Department, Faculty of Medicine, Jazan University, Jazan 45142, Saudi Arabia; hjesudoss@jazanu.edu.sa; 2 Faculty of Medicine, Jazan University, Jazan 45142, Saudi Arabia; 3 Family and Community Medicine Department, Faculty of Medicine, Jazan University, Jazan 45142, Saudi Arabia; aaalharbi@jazanu.edu.sa

**Keywords:** physical activity, sedentary behavior, physical activity guidelines, awareness, knowledge

## Abstract

Background: Physical activity guidelines recommend that adults engage in aerobic exercise and activities that preserve or increase muscle mass. The primary aim of this study was to assess the awareness and knowledge of these guidelines among adults in the Jazan region of Saudi Arabia. A secondary objective was to examine the role of awareness and knowledge in the adherence to physical activity guidelines. Methods: In this cross-sectional observational study, 1018 participants were recruited through a self-administered online survey. Participants’ awareness and knowledge about physical activity guidelines were assessed using a prompted questionnaire. Physical activity levels and weekly energy expenditures were evaluated using the International Physical Activity Questionnaire. Results: The proportion of the participants who reported being aware of the guidelines was approximately 48%, whereas the proportion of the participants who correctly identified the guidelines for moderate-intensity physical activity was 38%. However, only 23% correctly identified the muscle strength guidelines. Those who were aware of the guidelines were most likely to meet the physical activity recommendations (OR = 2.03; 95% CI = 1.55–2.65). Participants who reported being aware of the guidelines exhibited a significantly higher energy expenditure, measured in MET minutes per week (*p* < 0.01). Similarly, participants who correctly identified the guidelines had greater energy expenditure (*p* = 0.03). Conclusions: We found that adults in Jazan have a moderate level of awareness and knowledge of PA guidelines and that awareness is a predictor of adherence to these guidelines. Individuals who are aware of PA guidelines or have knowledge of them tend to have higher levels of physical activity. These findings suggest that public health campaigns that promote awareness and knowledge of the physical activity guidelines may accelerate the progress in engaging the Saudi population with these guidelines.

## 1. Introduction

Physical activity (PA) is well recognized for its role in health promotion and disease prevention [1]. Conversely, physical inactivity and sedentary behavior are linked with several chronic diseases and their risk factors [2]. Increasing evidence of the importance of PA to health has led to the establishment of PA guidelines to increase public knowledge about the minimum levels of PA required to improve health. These guidelines aim to enhance public knowledge, which could be reflected in PA behaviors. The PA guidelines for Americans [3], released in 2018, and the World Health Organization (WHO) guidelines on PA and sedentary behavior, released in 2020 [4], recommend at least 150 min of moderate-intensity aerobic PA, 75 min of vigorous-intensity aerobic PA, or an equivalent combination of both per week. In addition, these guidelines also recommend muscle-strengthening activities on at least two days per week. National PA guidelines for the Saudi population, based on the WHO recommendations, were introduced in 2020 [5]. 

Despite the well-documented benefits of PA in reducing the risk of morbidity and mortality from chronic diseases, physical inactivity remains a significant public health concern in Saudi Arabia, where a high percentage of the Saudi population remains physically inactive [6]. In 2013, the healthcare costs resulting from physical inactivity were estimated to represent about 1.7% of the national healthcare expenditure [7]. Furthermore, the population attributable fraction (PAF) for all-cause mortality due to physical inactivity in Saudi Arabia has reached 18.4. This PAF is higher than the median values for the eastern Mediterranean region (12.5%) and the WHO regions (9.4%) [8]. 

Awareness and knowledge, while not the only factors in behavior modification, play essential roles in facilitating behavioral change [9,10]. When individuals know the recommended levels of physical activity and understand the associated health benefits, they may consider PA as an important health behavior. This recognition can motivate individuals to integrate PA into their daily lives, leading to healthier lifestyles and a reduced risk of non-communicable diseases [11]. Yet, only a few studies evaluated the awareness and knowledge of PA guidelines. For instance, Bennett et al. reported that only a third of US adults recognized the 1995 PA recommendations [12]. Another recent study revealed that only 3% of US adults knew about the 2018 guidelines recommending at least 150 min of moderate-intensity PA per week [13]. In Finland, while 40% of young adult men were aware of the PA guidelines, only 7% accurately identified the recommendations for moderate-intensity PA [14].

This study aimed to assess the awareness and knowledge of PA guidelines among adults in the Jazan region of Saudi Arabia. Since awareness and knowledge of PA guidelines may motivate individuals to become physically active, it is also of interest to identify the demographic factors associated with the awareness and knowledge of PA guidelines. Therefore, a secondary aim of this study was to identify any possible associations between sociodemographic factors and the awareness and knowledge of PA guidelines, and to determine if the levels of awareness and knowledge were predictors of whether an individual met the PA guidelines or not. 

## 2. Methods 

### 2.1. Study Design and Participants 

This study was conducted as an observational, cross-sectional online survey between January and March 2024. We targeted adults aged 18 years and above in the Jazan region of Saudi Arabia. The Jazan province is one of Saudi Arabia’s 13 provinces located in the southwestern border of Saudi Arabia and has a population exceeding 1.6 million, as reported by the 2019 census from the Saudi General Authority of Statistics [15]. Participants were recruited online using snowball sampling recruitment methods, where individuals who received the study invitation link were asked to pass it on. Using power analysis, we calculated that we need 385 participants to achieve a 95% confidence level with a 5% margin of error and a population proportion assumption of 0.05. To enhance this study’s statistical power, the sample size was subsequently increased to 1018 participants.

Respondent demographic characteristics included sex (male or female), age (17–34 y; 35–49; 50–64), education level (bachelor’s degree and higher; some college; high school graduate or lower), marital status (single; married; divorced/widowed), household income (≤SAR 5000; SAR 5000–9999; SAR 10,000–14,999; SAR 15,000–19,999 or ≥SAR 20,000), job (employed; unemployed; student; retired), and living environment (urban, rural).

Other respondent characteristics included body mass index (BMI) category and PA level. BMI, calculated from self-reported height and weight, was categorized as underweight/normal weight (BMI < 25 kg/m^2^), overweight (BMI 25 to <30 kg/m^2^), and obese (BMI ≥ 30 kg/m^2^).

### 2.2. Physical Activity Assessment 

For the evaluation of participants’ PA levels, we used the short form of the International Physical Activity Questionnaire (IPAQ). The IPAQ is a validated questionnaire that includes questions about the duration and frequency of walking, moderate-intensity PA, and vigorous-intensity PA. Intensity is measured in metabolic equivalents (METs), which reflect the energy expenditure of various activities [16]. A MET is equal to an oxygen consumption rate of 3.5 mL/kg/min, representing the average oxygen uptake at rest [17]. Activity intensities are classified as follows: light (1.5–3 METs), moderate (3–6 METs), and vigorous (>6 METs) [18]. For IPAQ analysis, activities were categorized into walking (3.3 METs), moderate exercise (4 METs), and vigorous exercise (8 METs) [19]. Participants reported the frequency (days per week) and duration (minutes per day) of PA they engaged in for each activity level. To quantify physical activity in MET minutes per week, the following formula was applied: total MET-min/week = (walking METs × minutes × days) + (moderate METs × minutes × days) + (vigorous METs × minutes × days). PA levels of the participants were also reported as a categorical outcome—category 1 (low), category 2 (moderate), and category 3 (high)—according to the IPAQ scoring protocol. More specifically, the high PA category involves vigorous physical activity, achieving at least 1500 MET min/week or any combination of activities and totaling at least 3000 MET min/week. A moderate PA level involves vigorous activity for 20 min on three days or moderate activity for 30 min on five days, totaling at least 600 MET min/week. PA levels that do not meet the criteria for either moderate or high PA levels are classified as low PA. A “low” classification via the IPAQ indicates a failure to achieve the World Health Organization’s (WHO) recommended threshold of at least 150 min of moderate-intensity PA or 75 min of vigorous-intensity PA per week [4]. On the other hand, those categorized as engaging in ‘moderate’ or ‘high’ levels of physical activity would meet these recommendations.

### 2.3. Awareness and Knowledge of PA Guidelines 

The participants’ awareness of the national PA guidelines was assessed with the question, “Have you seen, heard, or read anything about governmental PA guidelines?”. The response choices provided were “Yes”, “No”, and “I don’t know”. Those who answered “Yes” were categorized as being aware of the guidelines.

Knowledge of the aerobic guideline for the adults was assessed with the question, “What is the recommended minimum minutes per week of moderate-intensity physical activity based on the national PA guidelines?” Response options included “60 min”, “90 min”, “150 min”, “210 min”, “410 min”, and “I don’t know”. Respondents who selected “150 min” were considered to have knowledge of the adult aerobic PA guidelines for achieving a moderate-intensity PA level. 

Knowledge of muscle strength recommendations (i.e., strength training) was assessed with the following questions: “What is the recommended minimum number of days for engaging in muscle-strengthening activities, according to the national physical activity guidelines?” The response options were “one time a week”, “two times a week”, “three times a week”, “four times a week”, “five times a week”, and “I don’t know.” Respondents who selected “two times a week” were considered to be aware of the muscle strength recommendations.

The awareness and knowledge questions above are linked to the broader construct of the awareness and knowledge of the PA guidelines and have been used previously [13]. The awareness question indicates whether participants were exposed to information about the guidelines. The knowledge question, on the other hand, is more specific as it assesses participants’ understanding of the guidelines. By combining these questions, a broader understanding of participants’ awareness and knowledge of PA guidelines was achieved via capturing both their exposure to and understanding of the guidelines.

### 2.4. Statistical Analysis 

Data were analyzed with IBM SPSS Statistics 22.0.2.0. Descriptive statistics, including frequencies and percentages, were used to summarize demographic variables. Chi-squared tests were used to examine any associations between levels of awareness and knowledge and demographic factors. The Kolmogorov–Smirnov test revealed that the MET-min/week data were not normally distributed. Thus, the non-parametric Mann–Whitney U test was used to examine the differences in the MET min/week according to the awareness and knowledge of participants’ PA guidelines. Logistic regression modeling was used to identify predictors of meeting PA guidelines by estimating adjusted odds ratios (aORs) and 95% confidence intervals. The aOR quantifies the strength of association between each factor and adherence to the PA guidelines after accounting for potential confounders. *p*-value < 0.05 was considered statistically significant for all analyses.

## 3. Results

### Participants Characteristics

The background characteristics of the study participants are shown in Table 1. The study sample comprised 1018 individuals, with a slightly higher female proportion (53.9%). The majority of the participants were young adults, with 62.0% aged 17–34 years. BMI data indicated that most participants were of normal weight (42.6%). Over half of the participants held a bachelor’s degree or above (54.9%). Most participants were non-smokers (86.4%) and lived in urban areas (76.2%). Regarding PA levels, 44.3% of participants were categorized as having low PA levels, 43.5% as having moderate PA levels, and 12.2% as having high PA levels.

**Figure 1:**
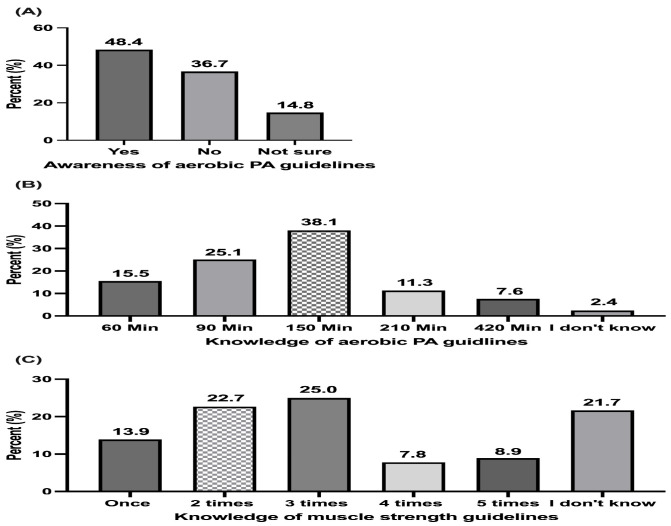
The percentage distribution of participants’ responses to the awareness of PA guidelines (**A**), knowledge of aerobic PA guidelines (**B**), and knowledge of muscle strength guidelines (**C**). (n = 1018).

Figure 1 shows participants’ awareness and knowledge of the PA guidelines. Nearly half of the participants reported being aware of the aerobic PA guidelines (Figure 1A). Regarding knowledge of the moderate-intensity aerobic PA guidelines, 38.1% correctly identified the minimum recommended duration of at least 150 min per week (Figure 1B). For muscle strength training, 22.7% knew the correct minimum frequency of two times per week (Figure 1C).

Table 2 shows the association of age, gender, BMI, and sociodemographic factors with the awareness and knowledge of the PA guidelines. Of the 1018 participants, 48.4% were aware of the physical activity (PA) guidelines, while 38.1% had knowledge of aerobic PA guidelines. Awareness and knowledge varied significantly with gender. While females demonstrated a higher awareness of PA guidelines at 54.3% compared to males at 41.6% (*p* < 0.01), they had less knowledge of aerobic PA guidelines (at 35.0% versus 41.8% for males (*p* = 0.02)). Higher awareness and knowledge were observed in participants aged 35–49 and those with a moderate PA level. Employment status and smoking were also associated with a higher awareness of the PA guidelines. No significant associations were found with respect to income, education, marital status, or living environment.

Binary logistic regression analysis (Table 3) revealed that participants’ awareness of PA guidelines significantly predicted their adherence to these guidelines. Participants who were aware of the PA guidelines were more likely to meet them (aOR 2.03, 95% CI [1.55–2.65]). In contrast, knowledge of the recommendations for aerobic PA or muscle strength training did not significantly predict meeting the PA guidelines. Females were less likely than males to meet the PA guidelines (aOR 0.54, 95% CI [0.40–0.74]). Living in a rural environment was associated with a lower likelihood of meeting the PA guidelines compared with living in an urban area (aOR 0.72, 95% CI [0.53–0.99]).

**Figure 2:**
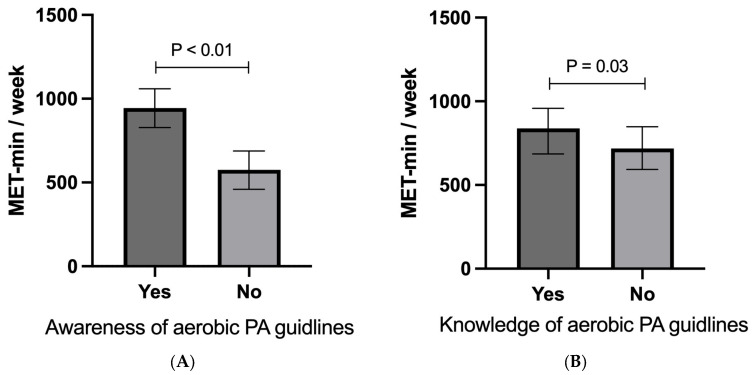
The weekly energy expenditure in MET min/week according to awareness (**A**) and knowledge (**B**) of PA guidelines. For awareness, ‘No’ and ‘Not Sure’ responses are combined under ‘No’. For knowledge, all responses other than “150 min/week” are merged into “No”. Each panel displays data as the median with 95% confidence interval. The Mann–Whitney U test was utilized to analyze differences between the groups that answered ‘Yes’ and ‘No’ within each category.

Figure 2 illustrates the energy expenditure in MET minutes per week according to participants’ awareness and knowledge of the PA guidelines. Panel A shows that participants who were aware of the PA guidelines exhibited a significantly higher energy expenditure (*p* < 0.01) than those who were not aware. Panel B indicates that individuals with the correct knowledge of the PA guidelines also exhibited a higher energy expenditure, though the difference was less pronounced (*p* = 0.03).

## 4. Discussion

In this study, we assessed the knowledge and awareness of PA guidelines among the adult population of the Jazan region in Saudi Arabia, and we examined the associations of BMI, gender, and demographic factors with the awareness and knowledge of the PA guidelines. A secondary aim of this study was to determine if the awareness and knowledge of the PA guidelines predicted adherence to these guidelines. Our findings revealed that a considerable portion of participants (48.4%) were aware of the PA guidelines, although fewer (38.1%) possessed specific knowledge about aerobic PA guidelines. To the best of our knowledge, this is the first national study to assess the awareness and knowledge of PA guidelines and muscle strength recommendations, leaving us without local benchmarks for comparison. However, with respect to aerobic PA guidelines, we found higher levels of awareness and knowledge than those reported in similar studies conducted in the USA and Finland [12,14,20].

Several policies and initiatives to promote PA have been introduced as part of the Saudi “Vision 2030” framework to create a vibrant society. Various Saudi agencies across different sectors, such as health and education, have either independently or in collaboration launched several initiatives to promote PA [21]. For instance, the initiative “Walk 30” initiated by the Ministry of Health, encourages members of the Saudi population to integrate PA into their daily routines. In the educational sector, this has been complemented by the promotion of PA among students by incorporating PA into their curricula [21,22]. Such efforts may eventually have helped to enhance the awareness and knowledge of the PA guidelines among the participants in this study. In addition, differences in assessment methods used to gauge awareness and knowledge of the PA guidelines may also have contributed to the high proportion of awareness and knowledge in this study. While we used a prompted questionnaire, which may lead to overestimating the true prevalence of correct knowledge, other studies have employed unprompted questionnaires, which tend to yield lower prevalence figures [12,13,23]. For instance, Cameron et al. showed that the prevalence of correct knowledge dropped significantly, from 37% to 4%, when responses from prompted and unprompted question formats, respectively, were compared [24].

Our study indicated that awareness, but not the knowledge of the PA guidelines, appears to be a significant predictor of meeting the PA recommendations. This finding may be attributed to the assessment methods used in our study. A simple yes/no question was used to assess awareness, which may have captured a broad proportion of participants being aware of the PA guidelines. This may have facilitated the strong association of awareness and meeting the PA guidelines observed in our study. Furthermore, while understanding PA recommendations is fundamental, environmental and social correlates are important to address when promoting a healthy lifestyle; therefore, an adequate knowledge of PA guidelines alone might not be sufficient to meet them when barriers to PA are not addressed [25].

Nevertheless, our results do suggest that greater levels of awareness and knowledge are associated with increased levels of energy expenditure. Prior research has suggested that people who meet the recommended levels of PA tend to be more informed about these guidelines [12,26]. This may indicate a link between the knowledge of physical activity recommendations and high PA levels, creating a positive loop in which increased knowledge leads to higher levels of PA. The relationship between the awareness and knowledge of the PA guidelines and PA levels may be bidirectional. On the one hand, individuals who have greater awareness and knowledge of PA guidelines may be more inclined to engage in higher levels of PA, as they understand the health benefits and have the information needed to implement these activities effectively. On the other hand, those who are already more physically active might seek out information to optimize their routines, thus gaining greater awareness and knowledge of the guidelines.

Interestingly, we found that awareness and knowledge significantly varied by gender, with females exhibiting higher awareness of guidelines but lower adherence to them, compared to males. This could be because females tend to have a higher health risk perception of adverse health conditions, such as cancer [27,28,29], which may prompt them to seek health knowledge, including PA guidelines, in order to counter their health risk perception and associated worries. Yet, our results indicated that the high level of awareness of aerobic PA guidelines did not translate into higher adherence to these guidelines when compared with males. This finding is consistent with previous national studies, which reported that females were consistently less active than males [30,31].

Previous studies have shown persistent sociodemographic disparities in which awareness and knowledge of the guidelines appeared to be higher with greater education and income [13,32,33]. However, we did not observe such a relationship in this study. This may be attributed, in part, to the relatively higher proportion of participants in our study who were aware and informed of the guidelines, thus mitigating the influence of sociodemographic factors, such as education and income. Furthermore, the unequal distribution of participants across different education and income levels may have impeded our ability to observe clear patterns of awareness and knowledge regarding PA guidelines. The majority of our participants had a bachelor’s degree and were in the lower-income group; this may have masked the variability needed to detect differences.

The pattern observed in the relationship between BMI categories and awareness of the PA guidelines is not linear. While one may expect that awareness would either increase or decrease steadily with BMI, our data showed a peak in awareness among the overweight group. This could be because overweight individuals are more receptive to public health messages. This observation is in line with the risk perception attitude framework, which suggests that a personal risk assessment significantly influences engagement in healthy behavior among obese individuals [34].

One strength of this study is its large sample size, which may enhance the generalizability of its findings and provide a solid foundation for our conclusions. Moreover, this is the first study that examined the knowledge of the Jazan’s adult population regarding the specific recommendations for both aerobic and muscle-strengthening activities. However, we acknowledge several limitations in our study. A single-item questionnaire may not accurately reflect the participants’ awareness and knowledge of the PA guidelines. Importantly, the unequal distribution of participants across age (62% were 17–34 years old) and the smaller representation of older age groups (35–49 and 50–64) might have affected the distribution of other sociodemographic factors, such as the level of education, occupation, and income. This may potentially impede the detection of possible significant differences in awareness and knowledge of the PA guidelines across different sociodemographic factors, such as education levels. Furthermore, the use of prompted questionnaires may have led to an overestimation of participants’ awareness and knowledge. This is because such methods can unintentionally guide responses toward more informed answers. Additionally, this study’s cross-sectional design limited our ability to establish cause-and-effect relationships. As a result, it is possible that our study findings could have been affected by selection biases. Another limitation is that we only assessed participants’ knowledge for moderate-intensity PA guidelines; no assessment was carried out for vigorous-intensity aerobic PA guidelines.

## 5. Conclusions and Future Directions

Our study revealed that 48% of the Jazan Saudi adult population were aware of the aerobic PA guidelines, with a lower proportion (38%) correctly identifying the guidelines for moderate-intensity aerobic PA. However, only 23% correctly identified the recommendation for muscle strength training guidelines. A gender disparity was observed regarding participants’ awareness of the PA guidelines, with females showing higher awareness compared to males. However, males demonstrated slightly higher knowledge of these guidelines than females. While only awareness of the PA guidelines emerged as a determinant in meeting these guidelines, both awareness and knowledge of the aerobic PA guidelines were associated with a higher energy expenditure. These results have implications for future government-led campaigns to promote PA by enhancing people’s awareness and knowledge of the PA guidelines. A multi-sectoral approach to enhance public awareness and knowledge of the PA guidelines via several ongoing initiatives may be a potential strategy to accelerate progress toward a healthier and more active society.

## Figures and Tables

**Table 1:** Participants characteristics.

Characteristics		n	%
Gender			
	Male	469	46.1
	Female	549	53.9
Age			
	17–34	631	62.0
	35–49	295	29.0
	50–64	92	9.0
BMI			
	Underweight	91	8.9
	Normal weight	434	42.6
	Overweight	305	30.0
	Obese	188	18.5
Education			
	Bachelor’s degree and above	559	54.9
	Some college	212	20.8
	Secondary school and lower	247	24.3
Marital status			
	Single	580	57.0
	Married	401	39.4
	Divorced/widowed	37	3.6
Job			
	Employed	413	40.6
	Unemployed	159	15.6
	Student	418	41.1
	Retired	28	2.8
Smoking			
	Smoker	92	9.0
	Non-smoker	880	86.4
	Ex-smoker	46	4.5
Income (SAR)			
	≤5000	554	54.4
	5000–9999	157	15.4
	10,000–14,999	130	12.8
	15,000–19,999	120	11.8
	≥20,000	57	5.6
Living environment			
	Urban	776	76.2
	Rural	242	23.8
PA level			
	Low	451	44.3
	Moderate	443	43.5
	High	124	12.2

Abbreviations: BMI, body mass index; SAR, Saudi riyal.

**Table 2:** Characteristics of the participants according to the awareness and knowledge of PA guidelines.

Characteristics		Awareness of PA Guidelines						Knowledge of Aerobic PA Guidelines					
		Yes n = 493 (48.4%)		No n = 525 (51.6%)		ES	*p*	Yes n = 388 (38.1)		No n = 630 (61.9)		ES	*p*
		n	%	n	%			n	%	n	%		
Gender	Male	195	41.6	274	58.4	**0.13**	**<0.01**	196	41.8	273	58.2	**0.07**	**0.02**
	Female	298	54.3	251	45.7			192	35.0	357	65.0		
Age	17–34	290	46.0	341	54.0	0.074	0.06	257	40.7	374	59.3	**0.08**	**0.04**
	35–49	160	54.2	135	45.8			105	35.6	190	64.4		
	50–64	43	46.7	49	53.3			26	28.3	66	71.7		
BMI	Underweight	41	45.1	50	54.9	**0.11**	**0.01**	33	36.3	58	63.7	0.07	0.21
	Normal weight	197	45.4	237	54.6			164	37.8	270	62.2		
	Overweight	172	56.4	133	43.6			129	42.3	176	57.7		
	Obese	83	44.1	105	55.9			62	33.0	126	67.0		
Education	Bachelor’s degree and above	261	46.7	298	53.3	0.06	0.19	207	37.0	352	63.0	0.04	0.51
	Some college	100	47.2	112	52.8			88	41.5	124	58.5		
	Secondary school and lower	111	51.4	105	48.6			93	37.7	154	62.3		
Marital status	Single	264	45.5	316	54.5	0.07	0.10	239	41.2	341	58.8	0.07	0.06
	Married	210	52.4	191	47.6			136	33.9	265	66.1		
	Divorced/widowed	19	51.4	18	48.6			13	35.1	24	64.9		
Job	Employed	210	50.8	203	49.2	**0.09**	**0.03**	148	35.8	265	64.2	0.08	0.09
	Unemployed	88	55.3	71	44.7			52	32.7	107	67.3		
	Student	185	44.3	233	55.7			178	42.6	240	57.4		
	Retired	10	35.7	18	64.3			10	35.7	18	64.3		
Smoking	Smoker	32	34.8	60	65.2	**0.09**	**0.02**	33	35.9	59	64.1	0.02	0.88
	Non-smoker	439	49.9	441	50.1			338	38.4	542	61.6		
	X-smoker	22	47.8	24	52.2			17	37.0	29	63.0		
Income (SAR)	≤5000	262	47.3	292	52.7	0.05	0.58	219	39.5	335	60.5	0.09	0.05
	5000–9999	71	45.2	86	54.8			51	32.5	106	67.5		
	10,000–14,999	68	52.3	62	47.7			49	37.7	81	62.3		
	15,000–19,999	64	53.3	56	46.7			39	32.5	81	67.5		
	≥20,000	28	49.1	29	50.9			30	52.6	27	47.4		
Living environment	Urban	381	49.1	395	50.9	0.02	0.44	299	38.5	477	61.5	0.02	0.60
	Rural	112	46.3	130	53.7			89	36.8	153	63.2		
PA level	Low	182	40.4	269	59.6	**0.15**	**<0.01**	160	35.5	291	64.5	**0.07**	**0.04**
	Moderate	246	55.5	197	44.5			169	38.1	274	61.9		
	High	65	52.4	59	46.6			59	47.6	65	52.4		
Knowledge (Resistance)	Yes	123	53.2	108	46.8	0.05	0.09	85	36.8	146	63.2	0.02	0.64
	No	370	47.0	417	53.0			303	38.5	484	76.8		
Knowledge (Aerobic)	Yes	206	53.1	182	46.9	**0.07**	**0.02**						
	No	287	45.6	343	54.4								

Abbreviations: ES, effect size; BMI, body mass index; SAR, Saudi riyal.

**Table 3:** Binary logistic regression for the factors predicting meeting the PA guidelines.

		Meet PA Guidelines
Variable		aOR (95% CI)
Gender		
	Male	Referent
	Female	**0.54 (0.40–0.74)**
Age		
	17–34	Referent
	35–49	0.67 (0.42–1.05)
	50–64	0.59 (0.30–1.15)
BMI		
	Underweight	1.13 (0.64–1.99)
	Normal weight	0.95 (0.65–1.34)
	Overweight	1.15 (0.78–1.70)
	Obese	Referent
Education		
	Bachelor’s degree and above	1.22 (0.88–1.71)
	In progress of completing bachelor’s degree but did not complete	0.78 (0.52–1.17)
	Secondary school	0.83 (0.36–1.88)
	Lower than secondary school	Referent
Marital status		
	Single	Referent
	Married	1.13 (0.73–1.75)
	Divorced/widowed	1.44 (0.67–3.14)
Job		
	Employed	Referent
	Unemployed	0.96 (0.60–1.54)
	Student	1.01 (0.67–1.67)
	Retired	1.76 (0.71–4.41)
Smoking		
	Smoker	Referent
	Non-smoker	0.91 (0.56–1.47)
	X-smoker	**2.43 (1.04–5.67)**
Income (SAR)		
	≤5000	Referent
	5000–9999	0.77 (0.50–1.18)
	10,000–14,999	0.85 (0.50–1.43)
	15,000–19,999	0.67 (0.39–1.15)
	≥20,000	0.52 (0.28–0.95)
Living environment		
	Urban	Referent
	Rural	**0.72 (0.53–0.99)**
Awareness of PA guidelines		
	Yes	**2.03 (1.55–2.65)**
	No	Referent
Knowledge of aerobic PA guidelines	Yes	1.11 (0.85–1.45)
	No	Referent
Knowledge of muscle strength PA guidelines	Yes	1.32 (0.95–1.79)
	No	Referent

Abbreviations: aOR, adjusted odds ratio; BMI, body mass index; CI, confidence interval. Note: Odds ratios shown in bold are significantly different from 1.0 (*p* < 0.05).

## Data Availability

The data that support the findings of this study are available from the corresponding author upon reasonable request.
